# Coordinated Expression of *FLOWERING LOCUS T* and *DORMANCY ASSOCIATED MADS-BOX*-Like Genes in Leafy Spurge

**DOI:** 10.1371/journal.pone.0126030

**Published:** 2015-05-11

**Authors:** Xinyuan Hao, Wun Chao, Yajun Yang, David Horvath

**Affiliations:** 1 Tea Research Institute, Chinese Academy of Agricultural Sciences; National Center for Tea Improvement, Key Laboratory of Tea Biology and Resources Utilization, Ministry of Agriculture, No. 9 south Meiling Road, Hangzhou, Zhejiang 310008, China; 2 United States Department of Agriculture, Agriculture Research Services, Bioscience Research Laboratory, 1605 Albrecht Blvd, Fargo, North Dakota, 58105, United States of America; 3 College of Horticulture, Northwest A&F University, Yangling, Shaanxi 712100, China; NARO Institute of Fruit Tree Science, JAPAN

## Abstract

Leafy spurge (*Euphorbia esula* L.) is a noxious perennial weed that produces underground adventitious buds, which are crucial for generating new vegetative shoots following periods of freezing temperatures or exposure to various control measures. It is also capable of flowering and producing seeds, but requires vernalization in some cases. *DORMANCY ASSOCIATED MADS-BOX* (*DAM*) genes have been proposed to play a direct role in the transition to winter-induced dormancy and maintenance through regulation of the *FLOWERING LOCUS T* (*FT*) gene, which also is likely involved in the vernalization process. To explore the regulation of *FT* and *DAM* during dormancy transitions in leafy spurge, the transcript accumulation of two previously cloned *DAM* splice variants and two different previously cloned *FT* genes was characterized. Under long-photoperiods (16 h light), both *DAM* and *FT* transcripts accumulate in a diurnal manner. Tissue specific expression patterns indicated the tissues with high *DAM* expression had low *FT* expression and vice versa. *DAM* expression is detected in leaves, stems, shoot tips, and crown buds. *FT* transcripts were detected mainly in leaves and flowers. Under dormancy inducing conditions, *DAM* and *FT* genes had an inverse expression pattern. Additionally, chromatin immunoprecipitation assays were performed using DAM-like protein specific antibodies to demonstrate that DAM or related proteins likely bind to cryptic and/or conserved CArG boxes in the promoter regions of *FT* genes isolated from endodormant crown buds. These results are consistent with the hypothesis that DAM proteins play a crucial role in leafy spurge dormancy transition and maintenance, potentially by negatively regulating the expression of *FT*.

## Introduction

Leafy spurge (*Euphorbia esula* L.) is an invasive perennial weed that infests range and recreational lands in the great plains of the US and Canada. The perennial nature of leafy spurge is mainly attributed to vegetative shoot growth from an abundance of underground adventitious buds that transition through well-defined phases of seasonally-induced dormancy [1]. Generally, adventitious buds are produced on the crown and lateral roots of leafy spurge in late May to early June and enter a state of paradormancy once formed. These buds will initiate new shoot growth when aerial tissues are destroyed or removed [2]. In the fall, environmental cues perceived by the plant (reduced temperature and photoperiod) induce adventitious buds to transition from paradormancy to endodormancy, which induces a state of extended growth incompetence [1]; meaning these adventitious buds are inhibited from resuming normal growth when exposed to growth-conducive conditions. Growth competency can be restored to these endodormant buds by extended cold temperature [3]. In leafy spurge, endodormancy is relatively shallow with some buds showing little to no dormancy, but most buds have greatly delayed growth or sluggish growth upon return to growth-conducive conditions following endodormancy induction [1], [3]. However, if the conditions remain cold, the buds will stay, non-growing, in an ecodormant state. Interestingly, endodormancy formation in the fall is required for leafy spurge to vernalize, and flowering competence gained by vernalization coincides with the transition from endodormancy to ecodormancy [1], [3]. These results support the hypothesis that there is an overlap in signaling mechanisms regulating dormancy and flowering [4].

FLOWERING LOCUS T (FT), a universal promoter of flowering, plays a crucial role in mediating the onset of flowering [5]. In arabidopsis (*Arabidopsis thaliana* Heyn) and other annual models, CONSTANS (CO), together with FT, GIGANTEA (GI), CRYPTOCHROME 2/CRYPTOCHROME-INTERACTING BASIC-HELIX-LOOP-HELIX (CRY2), PHYTOCHROME INTERACTING FACTOR 4 (PIF4), and the mediator complexes are important positive regulatory factors in *FT* transcriptional regulation, whereas FLOWERING LOCUS C (FLC), SHORT VEGETATIVE PHASE (SVP), TEMPRANILLO 1 (TEM1) and SCHLAFMUTZE (SMZ) are negative regulatory factors to balance *FT* transcription [6], [7], [8], [9], [10], [11], [12], [13], [14]. *FT* is primarily expressed in phloem companion cells in the leaves and the resulting protein is transported to the meristem where it initiates a transition of the vegetative meristem to a floral meristem [15]. The CO/FT regulatory module plays a vital role in controlling the photoperiod regulation of flowering in arabidopsis and, surprisingly, this module also controls short-photoperiod-induced growth cessation and bud set in poplar [14], [16]. Thus, it is assumed that perennial plants may share a similar signaling pathway to regulate other complex developmental or biological processes.

Since first associated with endodormancy, following the cloning of the *EVERGROWING* locus in peach [17], [18], *DORMANCY ASSOCIATED MADS-BOX* (*DAM*) genes have been identified from numerous woody plants. Meta-analysis of microarray data comparing different stages of bud dormancy in leafy spurge, raspberry (*Rubus idaeus*), poplar (*Populous* ssp.), potato, and grape (*Vitis vinifera*) [19] identified several MADS-box transcription factors related to the *DAM* genes from peach. Interestingly, *DAM* genes encoded one of the few differentially-regulated transcription factors identified in leafy spurge and 3 or more of the other species. In addition, the previous study also indicated that expression of two *DAM* transcripts was significantly increased in crown buds during the transition from paradormancy to endodormancy. One of the *DAM* transcripts (*DAM1*) was primarily expressed during endodormancy, and the other (*DAM2*) was primarily expressed in late endodormancy and during ecodormancy [19]. Subsequent studies indicated that both transcripts are splice variants from the same gene [20].

*DAM* genes have similarity to the transcription factors *SVP* and *AGAMOUS-LIKE 24* (*AGL24*) in *Arabidopsis* and have expression patterns consistent with a role in dormancy regulation in many perennial plants [18]. In arabidopsis, SVP negatively regulates *FT* by binding a CArG motif to maintain plants in vegetative growth [10]. Transcription of *FT* was coordinately down-regulated upon induction of *DAM*, which was paralleled by transitions from paradormancy to endo- and eco-dormancy in leafy spurge crown buds [19]. Further, over-expression of leafy spurge *DAM* genes in arabidopsis had a negative effect on flowering and growth [21]. Likewise, over expressing a Japanese apricot *DAM* gene in poplar enhances dormancy induction in poplar, and the highest *DAM* expressing lines had lower levels of *FT* transcripts than lines showing low *DAM* expression or which over-expression had little impact on the phenotype [22]. Therefore, because of the structural similarity between DAM and SVP, and the role of *FT* in controlling bud dormancy in poplar as well as the impact of expression of *DAM* genes on *FT* transcript accumulation in heterologous systems, the leafy spurge DAM proteins have been proposed to play a direct role in dormancy induction and maintenance through negative regulation of *FT* [19]. Subsequently, two genomic BAC clones containing *FT*-like genes from leafy spurge and several BAC clones containing very similar *DAM* gene have been cloned and characterized [20].

Here we test the hypothesis that DAM regulates *FT* expression, and document the expression of *DAM* and *FT* genes, including diurnal expression, tissue-specific expression, and the expression pattern under dormancy induction. Results indicate that *DAM* and *FT* genes are expressed in a manner consistent with DAM negatively regulating *FT* expression, but the minimal tissue-specific expression of *FT* in the buds along with minimal differential expression of DAM in the leaves suggests that modification of the hypothesis may be in order. Additionally, we examine the possible interaction of DAM with FT promoter elements using chromatin immunoprecipitation assays using an antibody proven to interact with the leafy spurge DAM protein(s).

## Materials and Methods

### Plant materials and treatments

Three-month-old leafy spurge plants, grown in sunshine mix in 1- × 8-inch conetainers under 16 h of natural and supplemental lighting (from 6:00 AM to 10:00 PM CST) and an average greenhouse temperature of approximately 25°C, were used in this study. For tissue-specific gene expression analysis, different organs were collected separately at 9:00 PM and frozen immediately in liquid N_2_. Every sample was harvested from five individual plants for each of three biological replicates. For diurnal gene expression analysis, mature leaves for each sample were collected from three individual plants every 4 hours started at 2:00 AM and continuing for three consecutive days. All samples were immediately frozen in liquid N_2_ and stored at -80 C. For dormancy induction analysis, plants were moved into a growth chamber under long-photoperiod (16 hrs) with cold night, morning, and evening (11 C for 16 hrs) but warm mid-day (24 C for 8 hrs) conditions. These conditions have been shown to induce endodormancy within 6 weeks [21]. Samples were collected at 0–8 days, and 1.5 through 7 weeks. Shoot apices and leaves were collected for expression analysis during the first four weeks of dormancy inducing conditions when crown buds were small. After that, leaves and crown buds were collected because aboveground parts stopped growing but crown buds were enlarged. Two biological replications were collected. Samples were frozen in liquid N_2_ immediately after collection and stored at −80°C for further study. After 7 weeks of treatment to induce endodormancy, plants were exposed to 5°C and short-photoperiod (8 hours) to vernalize and induce ecodormancy. After four weeks, we removed all aerial tissues and moved these plants into a greenhouse to check dormancy status (the plants with 20 weeks vernalization were also examined to confirm they had broken endodormancy). The length of the longest shoots from crown buds of at least 7 plants were measured after two weeks.

### RNA extraction and expression analysis

Frozen samples were ground in liquid N_2_ and RNAs were extracted following the pine tree extraction protocol [23]. The concentration of RNA was measured using a NanoDrop 1000 Spectrophotometer (Thermo Fisher Scientific, USA), and the quantity and integrity was determined by gel electrophoresis. RNA (5 μg) was first treated with DNase I (Invitrogen #18068–015) and then used for first-strand cDNA synthesis (Invitrogen #18080–051). qRT-PCR was performed using cDNA templates on an LightCycler 480 II (Roche, USA) Real-Time PCR system following the methods of Chao et al. [24]. Gene specific primers (S1 Table) were designed for quantifying transcript abundances, and the *SAND* gene (gene ID: CV03067B2A12.f1) was used as an internal control. *SAND* gene was verified to be stably expressed during bud development [24].

### DAM antibody preparation and immunoblot analysis

A conserved peptide (RRGLFKKAHELS) was designed for antibody preparation based on protein sequences generated from leafy spurge *DAM1* and *DAM2* (gene ID: EU339320.1). Peptide synthesis, antibody production, and affinity purification were carried out by Affinity Bioreagents (Golden, CO) according to the company’s general protocols. A pGEX-DAM fusion protein was generated for immunoblot analysis. pGEX-DAM fusion construct, protein overexpression, and affinity purification were performed following the procedures described [25]. Immunoblot analysis was done as previously described [26].

### Chromatin immunoprecipitation (CHIP) analysis

Frozen crown buds (collected from field-grown plants in Fargo, ND, USA on December 4^th^, 2012) were cross-linked under vacuum for 30 min in 50 ml formaldehyde solution (0.4 M sucrose, 10 mM Tris-Cl [pH 8.0], 1 mM EDTA, 1 mM PMSF, 20 μM MG132, 1% formaldehyde) at 4°C. Five ml of 1 M glycine was added and incubation was continued for an additional 5 min. Cross-linked buds were quickly rinsed with 20 ml distilled H_2_O_2_ and briefly dried on paper towels, then frozen in liquid N_2_. For one sample, 0.3 g of buds was ground in liquid and resuspended in 1.8 ml nuclei isolation buffer (0.25 M sucrose, 15 mM PIPES [pH 6.8], 5 mM MgCl_2_, 60 mM KCl, 15 mM NaCl, 0.9% Triton X-100, 1mM PMSF, 1 tablet protease inhibitor cocktail- cOmplete from Roche Life Science, cat.# 04693116001) and incubated on ice for 15 min. The sample was centrifuged at 14,500 rpm at 4°C for 10 min and the resulting pellet was resuspended with 800 μl lysis buffer (50 mM HEPES [pH7.5], 150 mM NaCl, 1 mM EDTA, 1% Triton X-100, 0.1% Na deoxycholate, 0.1% SDS, 1mM PMSF, 1 tablet protease inhibitor). Samples were sonicated on ice 8 times, 15 seconds each at 40% duty cycle and 20% power (BRANSON, Digital Sonifier). The majority of the resulting DNA fragments ranged in size from 200 to 500 base pairs (see S1 Fig). Sonicated samples were centrifuged at 14,500 rpm at 4°C for 5 min and the supernatant was transferred to a new Eppendorf tube. Equilibrated protein A-agarose beads (40 μl) was added for pre-clearing by rotating at 4°C for 1 hour. Beads were pelleted by centrifugation at 12,000xg at 4°C for 30 seconds. The supernatant was transferred to an Eppendorf tube with 10 μg of DAM1 antibody, with no antibody, or with 10 μg of an antibodies to a leafy spurge cyclin dependent kinase activating kinase [27] as negative controls. After overnight incubation with rotation at 4°C, 40 μl protein A-agarose beads were pre-absorbed with sheared salmon sperm DNA followed by rinsing 3 times with 1 ml fresh dilution buffer (1.1% Triton X-100, 1.2 mM EDTA, 16.7 mM Tris-HCl [pH 8.0], 167 mM NaCl). The pre-absorbed beads were added and another 2 hour’s incubation was performed. The beads were collected by centrifugation at 3,800xg for 30 seconds and washed in turn by 1 ml low salt wash buffer (150 mM NaCl, 0.1% SDS, 1% Triton X-100, 2 mM EDTA, 20 mM Tris-HCl [pH 8.0]), 1 ml high salt wash buffer (500 mM NaCl, 0.1% SDS, 1% Triton X-100, 2 mM EDTA, 20 mM Tris-HCl [pH 8.0]), 1 ml LiCl wash buffer (0.25 M LiCl, 0.5% NP-40, 0.5% deoxycholate sodium salt, 1 mM EDTA, 10 mM Tris-Cl [pH 8.0]) and 2 × 1 ml TE buffer (10 mM Tris-HCl [pH 8.0], 1mM EDTA). Finally, beads were washed twice with a total of 250 μl elution buffer (1% SDS and 0.1 M NaHCO_3_) with rotation at room temperature for 45 min. After brief centrifugation, the immune-complexes in supernatant were disrupted by overnight incubation at 65°C after adding 10 μl 5 M NaCl, then 5 μl 0.5 M EDTA, 10 μl 1 M Tris-HCl (pH 6.5) and 1 μl 20 mg/ml proteinase K followed by another 1.5 hour incubation at 45°C to degrade residual proteins. Immunoprecipitated DNA was extracted using QIAquick PCR purification kit (QIAGEN, USA) and eluted with 60 μl H_2_O. qRT-PCR was performed to verify the CHIP results using specific primer pairs (S1 Table).

## Results

### Diurnal expression of DAM and FT genes in leafy spurge

**Fig 1:**
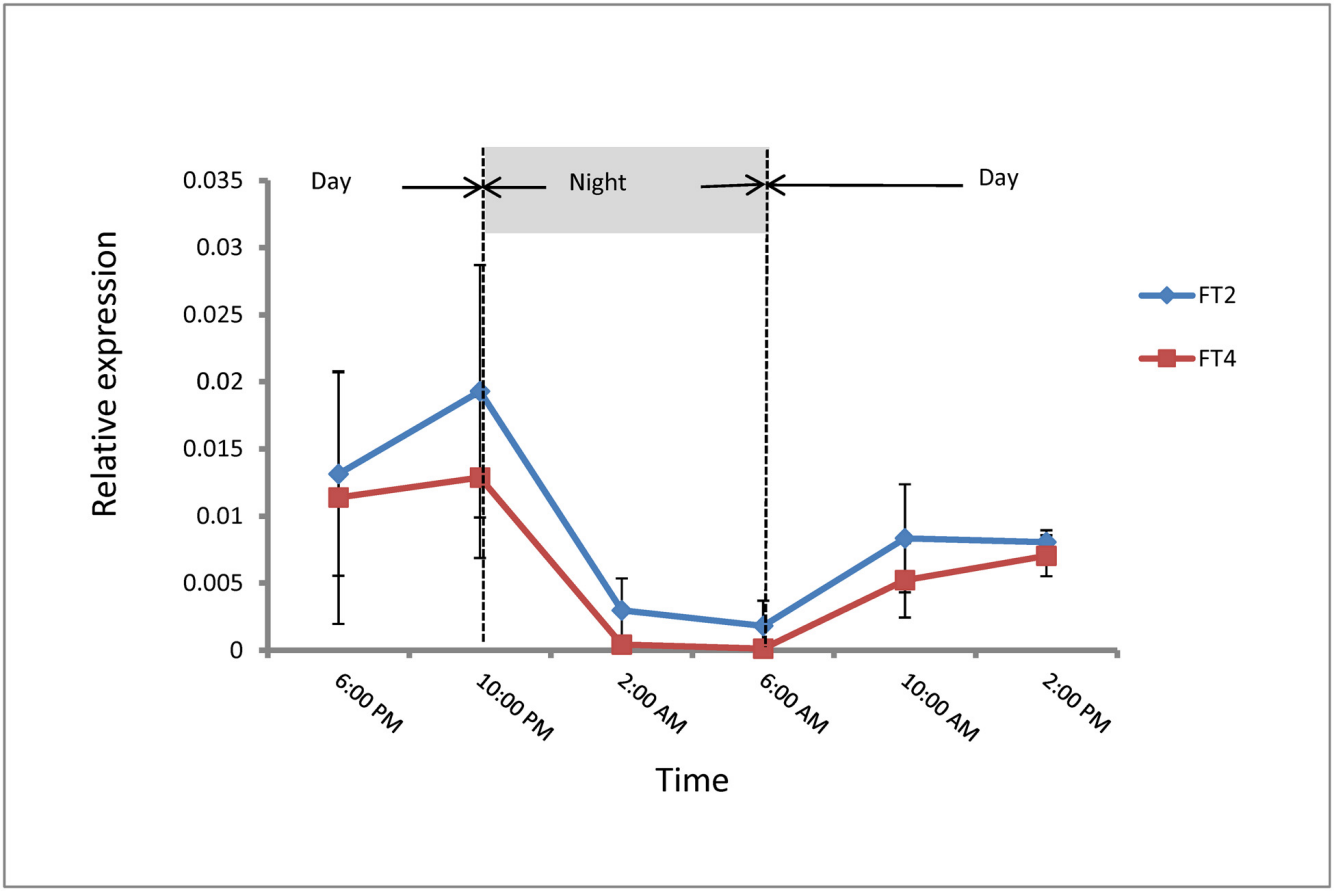
Average diurnal expression of *DAM* and *FT* genes under long-photoperiod condition. (A) *DAM1* and *DAM2* diurnal expression. (B) *FT2* and *FT4* diurnal expression. Plants were grown in greenhouse with 16 hours light (from 6:00 AM to 10:00 PM) with average temperatures of approximately 25°C. Gene expressions was examined in leaf tissue every 4 hours for three consecutive days. Error bars show standard deviation of the average of all three days for each time point.

*DAM* and *FT* expression has not been well characterized in leafy spurge. To determine the relationship between them we first investigated their diurnal and tissue-specific expression patterns under long-photoperiod conditions for three consecutive days (Fig 1). The results are consistent with both DAM transcripts having a diurnal expression patterns that peak between 2:00 PM and10:00 PM in the evening- prior to the dark period (Fig 1A). After that time point, accumulation of the two *DAM* transcripts decreased significantly throughout the dark phase and reached the lowest expression levels in at or about the time of daybreak (6 AM).

The expression level of both leafy spurge *FT2* (gene ID: JX966353.1) and *FT4* (gene ID: JX966355.1) genes started to increase at 6:00 AM in the morning, and reached a peak between 6:00 PM and 2:00 AM (Fig 1B). After that the expression of *FT* genes decreased dramatically and reached the lowest point in the morning at the onset of the light period.

### Tissue-specific accumulation of DAM and FT transcripts in leafy spurge

**Fig 2:**
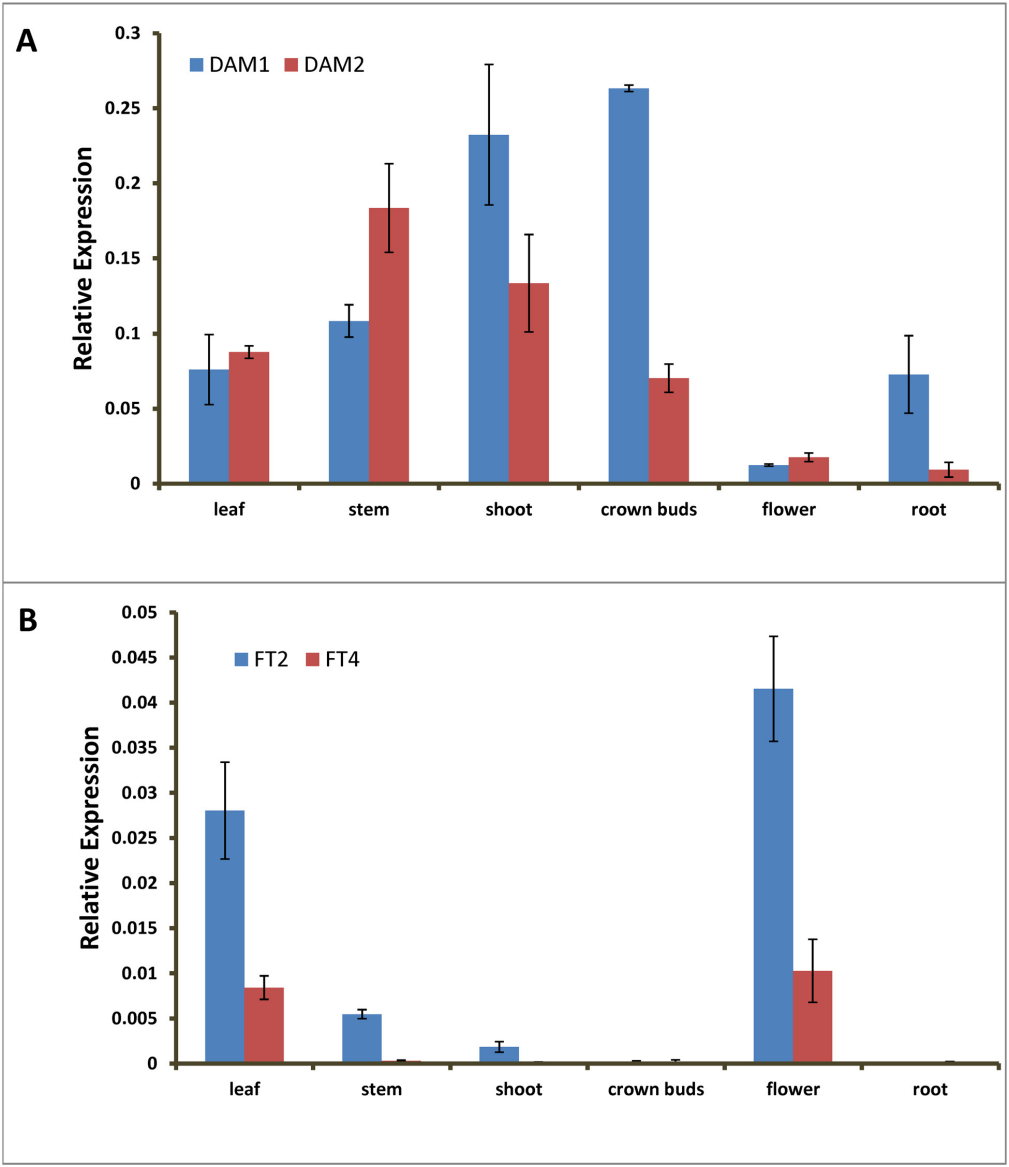
Tissue specific expression of leafy spurge *DAM* and *FT* genes. Gene expression in leaf, stem, shoot, crown buds, flower and root were examined at the end of a 16 hr photoperiod under non-dormancy inducing conditions. (A) *DAM1* and *DAM2* expression in different organs. (B) *FT2* and *FT4* expression in different organs. Error bars show standard deviation from three biological replicates.

The transcript levels for *DAM* and *FT* in different organs were detected at the end of the 16 hour photoperiod. The tissue specificity data indicated that transcript abundance for both *DAM1* and *DAM2* was relatively greater in leaves, stems, shoots and crown buds, and was less abundant in flowers (Fig 2). *DAM1* particularly accumulated mostly in shoots and crown buds and, in roots, transcript abundance for *DAM1* was significantly greater than that of *DAM2* (Fig 2A). Both *FT2* and *FT4* genes were preferentially expressed in leaves and flowers, and transcript abundance of *FT2* is greater than that of *FT4* in all measured tissues (Fig 2B).

### Long photoperiods with cold evening nights and mornings can induce endodormancy and vernalization increases growth potential of crown buds

**Fig 3:**
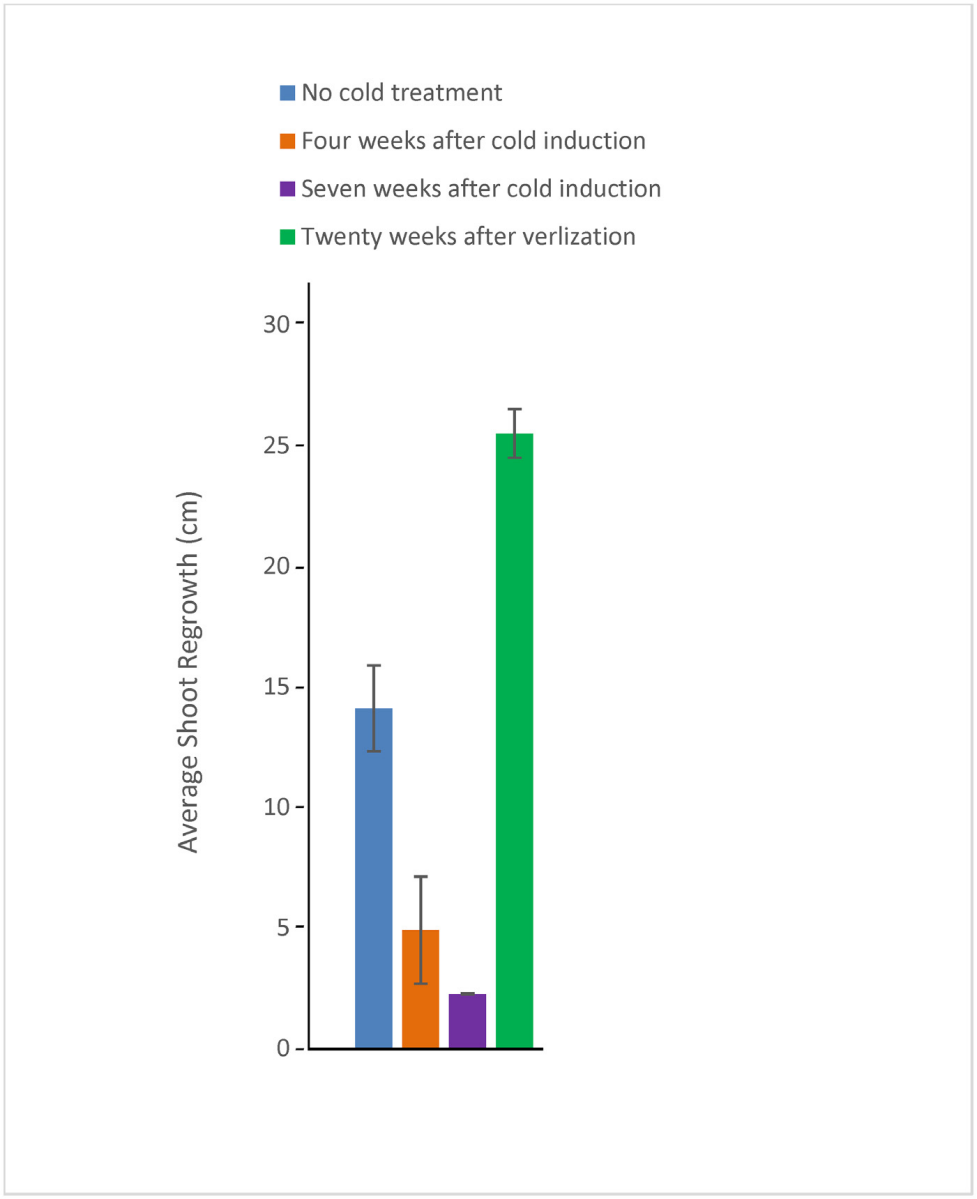
Comparison of the crown bud regrowth after 0, 4, or 7 weeks of endodormancy-inducing conditions. After the designated time points following dormancy induction or following vernalization, the aerial parts of the plants were removed, and the excised plants were grown in greenhouse for two weeks. Above chart only shows the average length of the longest shoots growing from crown buds after 0 through 7 weeks of endodormancy-inducing treatment and after twenty weeks vernalization. Error bars show range of the average of the longest stem from 7 plants for each experiment.

We confirmed that the treatment of long photoperiods with cold evenings, nights, and mornings would induce endodormancy, and that extended cold would not only release the plants from endodormancy, but increase the growth potential of the crown buds (Fig 3). After the decapitated plants with different treatments (long photoperiod no cold—paradormant, 4 weeks or 7 weeks of long photoperiod with cold evenings, nights and mornings—endodormant, and endodormant-inducing conditions followed by 20 weeks of short photoperiod cold days and nights- ecodormant/vernalized) were moved to greenhouse, the regrowth from crown buds was measured after 2 weeks (Fig 3). Regrowth was significantly suppressed from plants receiving four to seven weeks of endodormancy-inducing conditions relative to either paradormant buds that had received no cold treatment. In contrast, shoot growth from crown buds increased significantly after twenty weeks vernalization treatment and 100% of these plants flowered after three weeks in green house (data not shown). These results confirmed earlier studies that consistent long photoperiod with cold evenings, nights, and mornings caused adventitious buds to transition from paradormancy to endodormancy, whereas twenty week cold treatments resulted in a transition to ecodormancy and maximal growth competency.

### Correlating DAM and FT expression during endodormancy inducing and releasing conditions

**Fig 4:**
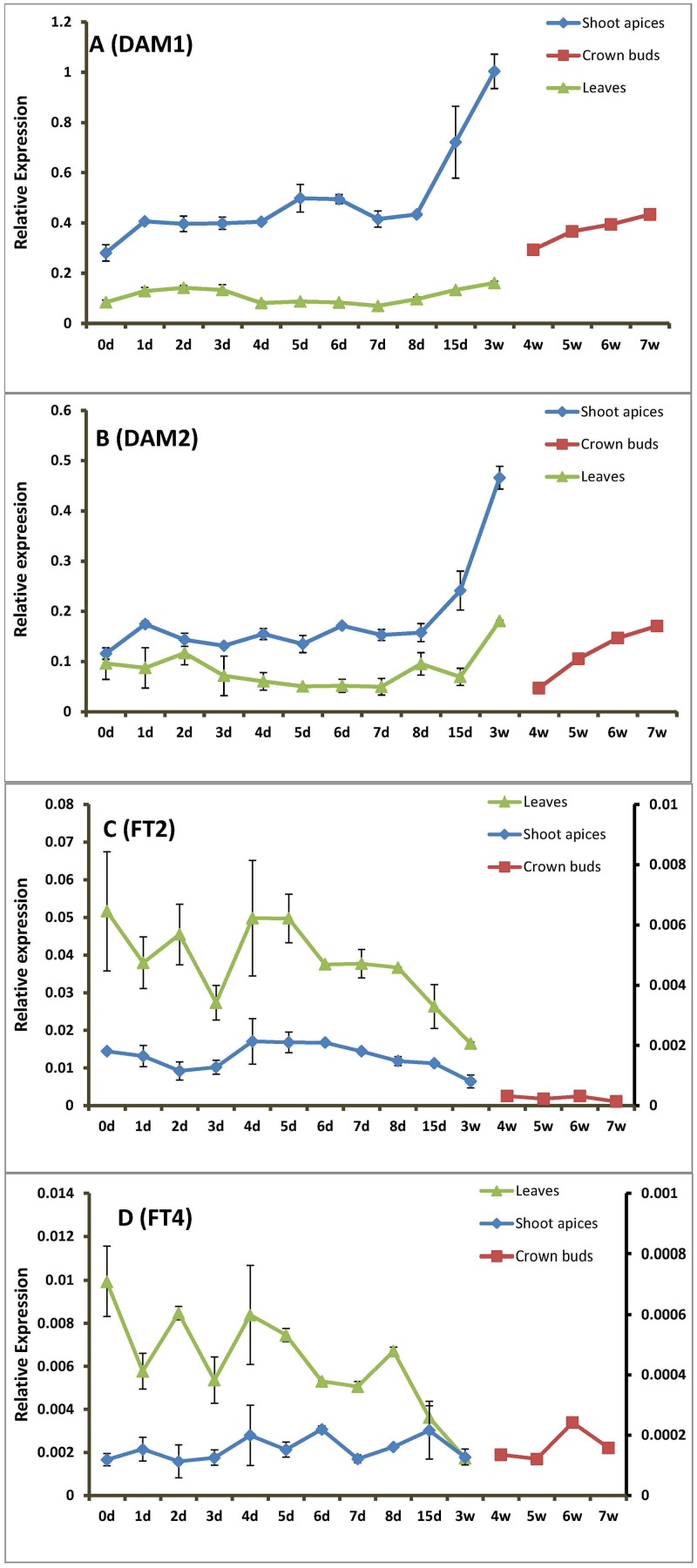
The average expression patterns of leafy spurge *DAM* and *FT*. Transcript levels for (A) *DAM1*, (B) *DAM2*, (C) *FT2*, and (D) *FT4* are shown during 7 weeks of endodormancy-inducing conditions. The plants were treated with long-photoperiod (16 hr) but cold nights, mornings and evenings (11°C for 16 hr) for endodormancy induction. The expression patterns of the four genes were examined at different duration of endodormancy induction in leaves and shoot apices/crown buds (in shoot apices from 0d to 21d, in crown buds from 4 weeks to 7 weeks). X-axis represents the length of dormancy induction (day (d), week (w)), and the right Y axis, when present, represents the expression of the *FT* genes in shoot apices/crown buds. Error bars show range from two replicates.

To identify the relationship in transcript abundance between *DAM* and *FT* gene during endodormancy induction, the transcript levels of *DAM* and *FT* were detected in leaves and shoot apices for seven weeks under endodormancy-inducing conditions (Fig 4). The expression data indicated that, *DAM1* and *DAM2* had similar accumulation patterns (Fig 4A and 4B); both *DAM1* and *DAM2* transcripts increased slightly but significantly during the first day, but increased substantially in shoot tissue between 8 and 15 d of treatment. However, by week 4 the shoot tips had ceased growing and were greatly reduced in size and leaf tissue had senesced. In crown buds (which were too small to sample early in the dormancy process), the levels of *DAM* transcripts increased sharply from the fourth week when crown buds were first collected through 7 weeks. In leaves, *DAM2* transcript had a weak increase in accumulation after two weeks of endodormancy-inducing treatment. However, no clear trend was detected in leaves for *DAM1* transcript accumulation.

*FT2* and *FT4* also had very similar transcript accumulation patterns (Fig 4C and 4D). Interestingly, accumulation of both *FT* transcripts was clearly suppressed in leaves after one week cold treatment, which was coordinately paralleled by increased *DAM* transcript accumulation in the shoot apices. Additionally, *FT2* transcripts were detected in shoot apices/crown buds at low levels and declined below initial levels to nearly undetectable levels beginning at 15d following endodormancy-inducing conditions.

### Antibody verification and CHIP assays

**Fig 5:**
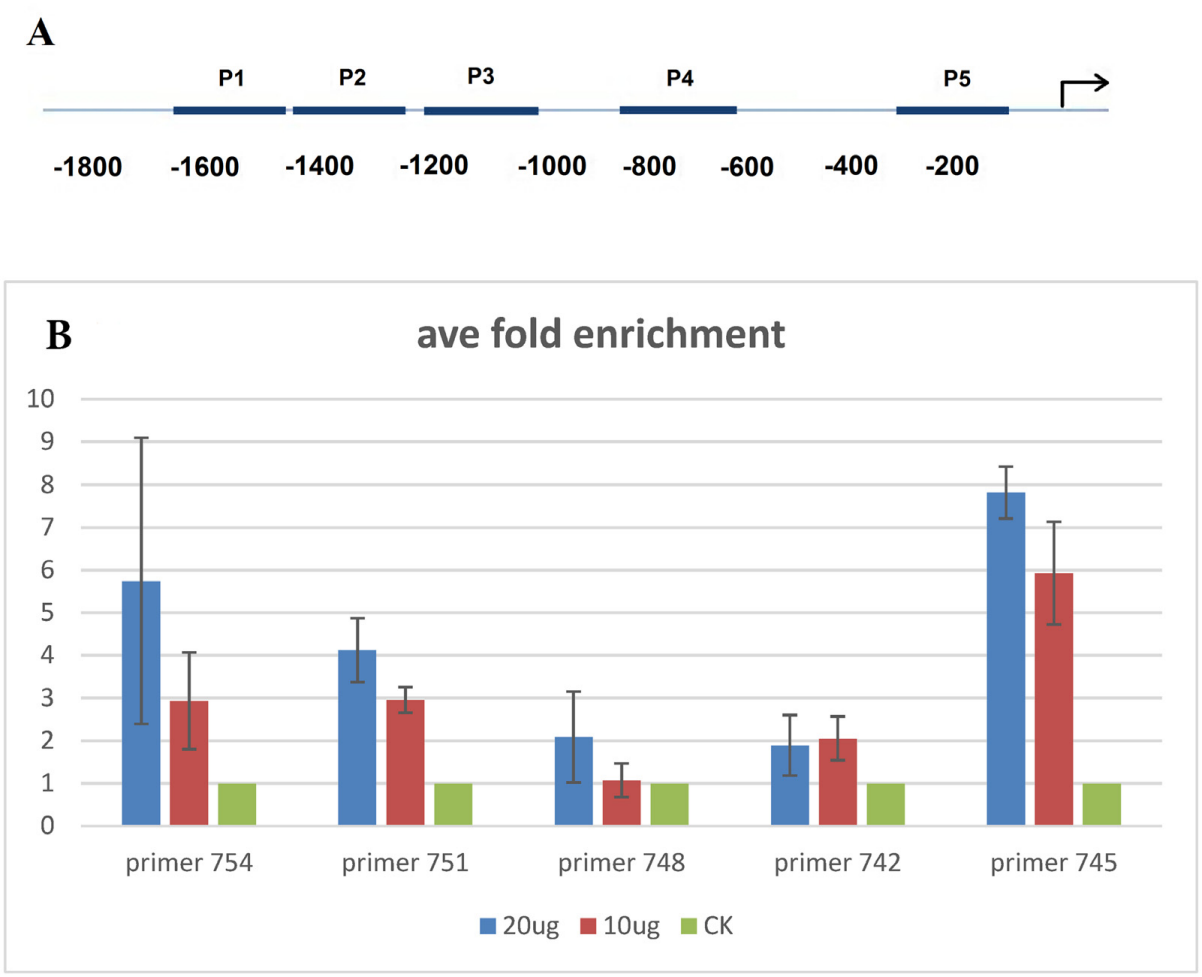
Chromatin immunoprecipitation (CHIP) analysis of leafy spurge *FT2* promoter using a leafy spurge DAM peptide antibody from endodormant crown buds. (A) The location of primer pairs (P1 to P5) in the *FT2* promoter region used in the qRT-PCR analysis of CHIP assays. (B) The average fold enrichment analysis of immunoprecipitated DNA fragments by qRT-PCR in CHIP assay with different amounts of antibody as noted. Error bars represent the range from two separate experiments.

Antigenicity of the DAM antibody was verified by immunoblot analyses using a GST-DAM fusion protein. The results revealed that DAM antibody correctly recognized the GST-DAM1 and GST-DAM2 protein (S2 Fig). Additionally, only two highly similar genes (or alleles of the same gene) have been found in leafy spurge through genomic and cDNA sequencing that would likely react with the antibody (S2 Table). Several other related genes were also identified that have amino acid sequences with varying degrees of similarity to the sequence that the antibody was designed to recognize (S2 Table). In an unrelated study, only four of these genes were shown to have transcripts that accumulated to relatively high levels in ecodormant buds. Based on these results, the potential for DAM regulation of *FT* was investigated by ChIP analysis using the DAM-binding antibody and endodormant crown buds when *DAM* was most highly induced, and *FT* was concomitantly down-regulated (Fig 3). Five primer pairs from the *FT2* promoter region were designed for quantitative PCR following ChIP (Fig 5A) and the precipitated DNA fragments were mainly enriched in regions 1, 2 and 5. In addition, greater quantities of DNA fragments were pulled down with higher doses of antibody (Fig 5B). However, under the same conditions, addition of greater than 30 μg antibody per sample did not increase the amount of precipitated DNA fragments (data not shown). It is clearly indicated that one or more DAM-like proteins binds the promoter region of *FT2* in crown buds during endodormancy. We analyzed the *FT2* promoter region and marked the putative conserved transcription factor binding sites. In region 1, an unknown but conserved putative binding site (tttcatgatgggtt) was found. This motif is also conserved in the promoters of leafy spurge *FT4* and poplar *FT2* (Potri.010G179700 with sequence tttcatgctgggtt). Two other putative CArG boxes were identified in region 2. No additional conserved or known regulatory sequences were identified in the DAM bound region covering the 3´ most fragment, but this region was less than 150 bases from the putative TATA box.

## Discussion

### DAM and FT may engage different regulatory networks under long-photoperiod conditions

Expression data indicated that *DAM* and *FT* transcript abundance appears to follow a similar diurnal rhythm. At least in the case of the *DAM* transcripts, there appears to be some indication that the expression patterns—particularly the fact that they appear to peak prior to the beginning of the night cycle, are consistent with a circadian response. *DAM* genes are proposed to be functional homologues to arabidopsis *SVP* genes [19]. In arabidopsis, *SVP* is negatively regulated by the circadian clock proteins LHY and CCA1 [10], [28], [29] and protein accumulation of SVP in arabidopsis [28] mirrors transcript abundance (Fig 1) of *DAM* in leafy spurge. It is unclear if *DAM* is also regulated by circadian clock associated proteins in perennial plants in a manner similar to *SVP* of arabidopsis. However, the presence of conserved evening elements in the promoters of leafy spurge *DAM* genes [19] is consistent with this possibility. Additional experiments with a shift from diurnal photoperiods to constant light or darkness will be required to determine if the response was the result of circadian regulation or not.

In arabidopsis, *FT* expression levels are influenced by the regulation of CONSTANS (CO) abundance [30]. Under long-photoperiod conditions, the transcript abundance of *CO* is regulated by the circadian clock [31] and its posttranscriptional regulation is triggered by light [32], [33], [34]. Therefore, in arabidopsis, the activation of *FT* transcription mainly depends on the light-mediated posttranscriptional regulation of *CO* [30]. In leafy spurge under long-photoperiods, *FT* expression levels also appear to follow the light status with peak expression at the end of the photoperiod with no apparent anticipation of a light or dark cycle. Thus, our observations would be consistent with a CO/FT regulatory module under long-photoperiod conditions controlling *FT* expression in leafy spurge, assuming that the products of these homologs perform similar function in leafy spurge as they do in arabidopsis. Overall, the diurnal expression patterns of *DAM* and *FT* in leafy spurge appear to be similar to the diurnal expression patterns or *SVP* and *FT* in arabidopsis.

### Expression of DAM and FT in leafy spurge are similar to that in other species

Tissue-specific gene expression studies indicated that the *DAM1* transcript is preferentially expressed in shoot apices and crown buds, and *DAM2* transcript is preferentially expressed in stems and shoot apices, with some expression in leaves (Fig 2). In numerous perennial species, *DAM* genes are highly expressed in the shoot apices [35] and references therein. Expression of all six tandem-arrayed *DAM* genes of peach are detectable in vegetative tissues, bud tissues, and with the exception of *DAM1*, also in floral organs [36]. Similarly, leafy spurge *DAM1* and *DAM2* transcripts were detected in most vegetative organs (Fig 2). These results suggest that *DAM* genes have no strict tissue specificity. However, the increased transcript abundance in shoot apices and crown buds and decreased transcript abundance in flowers, leaves, and stems suggest that *DAM* genes are preferentially expressed in the meristem or forming leaf tissues in leafy spurge as in other species.

Both *FT2* and *FT4* had greater transcript abundance in flowers and leaves; *FT2* also had significant transcript accumulation in stems and detectable transcript levels in shoot apices. FT plays a central role in mediating the onset of flowering, as a mobile signal, which is generally produced in leaves and vascular tissues and transported to the shoot apex [5]. In leafy spurge, the increased abundance of *FT* in leaves and flowers are consistent with a role for *FT* in flowering regulation, assuming that these homologs functions similarly in leafy spurge as it does in other species. In Lombardy poplar, *PnFT1* and *PnFT2* also had strong expression in early-summer leaves and capsules of reproductive trees, while *PnFT3/4* had strong expression in stems and winter lateral buds. Further, it is known that the two orthologues of *FT* genes, *FT1* (equivalent to *FT3/4* above) and *FT2 (*equivalent to *FT1* and *2* above), from poplar have different expression patterns and likely regulate flowering and vegetative growth differentially [37]. The lack of cold-induced expression for two leafy spurge *FT* genes is consistent with the expression pattern of the poplar *FT2* gene that controls vegetative growth. The combined sequence similarity of leafy spurge *FT2* and *FT4* with the conserved promoter element of poplar *FT2* but not *FT1* further suggests that the cloned leafy spurge *FT* genes most likely are functional orthologues of poplar *FT2*. However, functional confirmation in leafy spurge or other model perennial systems will be required to verify this hypothesis.

### Examination of gene expression evidence for regulation of FT by DAM

The lack of opposite diurnal expression patterns of *DAM* and *FT* is inconsistent with a direct role for DAM regulation of *FT*. Instead, both *DAM* and *FT* share common diurnal expression patterns and, thus, there may not be a direct regulatory relationship between the accumulations of their respective transcripts in regards to regulation of diurnal expression. However, in arabidopsis, where a direct regulatory pathway of *SVP* and *FT* genes has been established, both genes also appear to be similarly diurnally expressed with highest expression in the evening [10], [28], [29], [38].

The lack of accumulation of either *DAM* transcripts in leaf tissues following endodormancy-inducing conditions is problematic. Given the hypothesis that DAM regulates *FT* in leafy spurge the way SVP regulates *FT* in arabidopsis [21], we expected a strong increase in *DAM* expression coordinately with repression of *FT* expression in the leaves. It is unclear if the lack of *DAM* induction was due to the observed increasing senescence of the leaves after several weeks of endodormancy inducing conditions (data not shown), or if some other factors were responsible for the limited increase in *DAM* accumulation. However, a slight accumulation of *DAM1* transcript in the leaves appears to occur after 7d in leaf tissue following endodormancy induction and this precedes the significant drop in *FT* expression after 8d. Thus, it is possible that the repression of the *FT* genes under endodormancy-inducing conditions could be due to *DAM1* or *DAM2* expression. Likewise, in the shoot tips, *FT* transcript accumulation did not drop below its initial levels for nearly a week after the observed strong increase in *DAM* expression. Because we did not observe changes in FT expression prior to observing changes in DAM expression, these observations are consistent with the potential role for DAM negatively regulating *FT*.

As noted above, *DAM* is primarily induced and expressed in the buds and meristem (Fig 2). In arabidopsis, SVP is also accumulates in the meristem where it prevents flowering in part by inhibition of the induction of *SOC1* by FT [29] and by direct repression of *SUPPRESSOR OF CONSTANS 1* (*SOC1*) [10]. Variation of *SOC1* alleles has been associated with dormancy release in peach [39]. A *SOC1* homologue in strawberry (*Fragaria vesca*) was also shown to positively control vegetative growth [40]. *SOC1* was down-regulated in poplar following endodormancy induction [41]. Thus, it is also possible that DAM might also regulate endodormancy induction in leafy spurge through mechanisms similar to how SVP regulates flowering in arabidopsis. In support of this hypothesis, *SOC1* was down regulated by endodormancy-inducing conditions in leafy spurge in two separate microarray experiments [19], [42] and recently in an independent RNAseq experiment (unpublished), albeit not at our fairly strict significance cutoff of p<0.005.

Our results are, however, consistent with DAM having a potential negative regulatory role in *FT* gene expression in the leaves since the significant decrease in *FT* expression did not precede the significant increase in *DAM1* transcript accumulation. Likewise, previous experiments indicated that *FT* expression drops significantly during endodormancy and concurrently with an increase in *DAM* expression [21]. Also, although minimally expressed in the shoot tip (which includes young expanding leaves), *FT2* expression became undetectable following the increase in *DAM* expression. Additionally, other tissues with high *DAM* expression invariably had low *FT* expression (Fig 2). Although both *DAM* and *FT* transcripts accumulated in leaves under long-photoperiod conditions, the change in accumulation of *DAM* transcripts during endodormancy-inducing conditions was most evident in shoot apices and crown buds. Thus, although the coordinate expression of *DAM* and *FT* are consistent with a role in dormancy regulation, the fact that they are preferentially expressed in different tissues make it unclear if DAM directly regulates *FT* to induce endodormancy.

### Induction of *DAM* and repression of *FT* precedes endodormancy induction

Endodormancy was induced in crown buds with the exposure of whole plants to a combination of long-photoperiods with cold evening, night, and morning conditions (Fig 3). The complex mechanism of perceiving changes in temperature and photoperiod in perennial plants has been well discussed [3], [4], [18], [43]. Crown buds became fully endodormant after four to seven weeks of dormancy-inducing treatment, as shown by comparing regrowth from crown bud with treated (endodormant), control (paradormant), and vernalized (ecodormant) plants (Fig 4). Other studies have shown similar levels of leafy spurge growth inhibition in response to low temperatures and short photoperiods [1], [3], [42]. Both leafy spurge *DAM1* and *DAM2* transcripts showed some accumulation in shoot tips after the first day of endodormancy-inducing treatment followed by fluctuating RNA levels for approximate one week, and then a sharp and constant increase in accumulation one week after of treatment. The slope of the accumulation of *DAM* transcripts in crown buds is consistent with induction prior to the first collected time point at four weeks. The expression of leafy spurge *FT* genes was coordinately repressed when *DAM* genes were up-regulated- see above.

The one to two week delay prior to induced accumulation of *DAM* transcripts and repression of *FT* intimates a signaling mechanism requiring long term cold (longer than the short cold periods needed to induce many cold-regulated genes in arabidopsis [44]. Because temperature generally fluctuates in fall, a short cold period would not be a reliable signal for endodormancy initiation. In this study, the induction of endodormancy was not evident until somewhat later than the observed increase in *DAM*, and decrease in *FT* transcript accumulation. However, the earliest test for endodormancy induction was four weeks, and thus we cannot rule out the immediate establishment of endodormancy with induction of *DAM* and repression of *FT*. Thus, although our observations are consistent with these genes having a role in endodormancy induction, it is likely that other upstream and downstream genes or signaling processes may also be involved.

### DAM may regulate *FT* by binding a cryptic CArG box in promoter region

Because *DAM* and *FT* expression analysis was suggestive, but not convincing, we investigated the direct binding of DAM to the *FT* promoter using ChIP assays. Dose-dependent pull-down assays with leafy spurge DAM antibody indicated that leafy spurge DAM (or another very similar protein) interacts with the FT promoter, mainly with the regions 1 and 5 within the promoter of *FT2* (Fig 5). Thus, the DAM (or DAM-like) proteins likely directly bind to these regions in vivo, or interacts directly with other transcription factors that bind to these regions. In arabidopsis, SVP negatively regulates *FT* by binding to the CArG motifs in the promoter region [5], [38], and six CArG motifs were identified from 1.8-kb promoter region of *FT* and one from the first intron [45]. It was also demonstrated that the vCArG III/IV and vCArG V motifs were efficiently precipitated by SVP-HA antibody in ChIP assays [38]. In this study, regions 1, 2 and 5 of the leafy spurge *FT2* promoter have similar locations with variants of CArG III/IV and CArG V motifs in the *FT* promoter region of arabidopsis. A search of the *FT2* promoter region highlighted putative transcription factor binding sites and conserved motifs (S3 Fig). Interestingly, an unknown motif (tttcatgatgggtt) was found in region 1, which could represent an atypical CArG motif. Moreover, this motif is also conserved in similar regions of the leafy spurge *FT4* and poplar *FT2* promoters. Because CArG box sequences can be variable for SVP protein binding [46], we suggest that this conserved motif may serve as a novel CArG box that is recognized by the leafy spurge DAM protein. However, the region 2 was also enriched in the ChIP assays, and this region contains two CArG boxes, one of which (with the sequence cctattgg) is very similar to a CArG box in the same promoter region of the poplar *FT2* gene promoter (with the sequence cctcttgg) and which is identical in the first 6 bases to the CArgIII binding site in the arabidopsis *FT* gene.

It should be noted though, that only *DAM1/2* and three other *DAM*-like transcript shows high accumulation during ecodormancy (data not shown). Additionally, it has been proposed that DAM and other related proteins likely have additional targets besides *FT* [22]. We also agree that other targets (such as *SOC1*) might be viable targets for DAM and that such interactions have a potential role in dormancy responses. Consequently more work is needed to determine not only the additional targets of DAM proteins, but also the complex interactions of other potential regulators of dormancy on expression of *FT*. Towards this end, we have developed an antibody that recognizes the DAM protein and have data from chromatin immunoprecipitation assays that indicates it can specifically interact with chromatin that includes the conserved CArG box in the *FT* promoter. However, further studies are needed to confirm the specificity of this antibody and the specificity of the CArG box binding supported by these initial observations.

In conclusion, evidence here and in the literature strongly suggest the DAM/FT regulatory module may be a common mechanism controlling dormancy development in perennial plants. *FT* genes, as crucial developmental regulators, have multifaceted roles in plant development- including bud dormancy [16], [37], [47]. *FT* is regulated by both epigenetic mechanisms and by many regulatory factors or complexes binding to different elements in the promoter or introns of the *FT* gene [5]. DAM has similarity to other proteins known to negatively regulate *FT* in other systems. Leafy spurge *DAM* and *FT* genes have opposite seasonal expression patterns during para- to endo-dormancy transition in the fall [19]. Here we show that *DAM* and *FT* have expression patterns that generally consistent with the hypothesis that DAM negatively regulates *FT*. Additionally a conserved CArG box exists in the *FT* promoters of leafy spurge and poplar, and may serve as the binding sites for DAM or other very similar proteins. Further, conservation of the potential DAM binding site in poplar and leafy spurge suggests that the DAM/FT regulatory module may be a common mechanism controlling endodormancy induction in perennial plants. Collectively, these results provide further evidence for DAM having a direct role in negatively regulating *FT* in leafy spurge.

## Supporting Information

S1 Table List of primers. Primers used for qRT-PCR to determine gene expression and chromatin immunoprecipitation result analysis.
(DOCX)

S2 Table List of contigs with similarity to predicted DAM1 antibody binding site. List of transcripts assembled from three different expression analyses of leafy spurge including two containing dormant and growing bud samples that were identified as having sequence similarity (E values < 10E-5) with *DAM1* in a BlastX search of the transcriptome database. Highlighted contigs are from genes that have good levels of expression (> 100 transcripts per million) in ecodormancy.
(DOCX)

S1 Fig Agarose gel indicating size of the sonicated DNA used in the ChIP assays. Ethidium stained agarose gel showing the size range of the sonicated and precipitated DNA used for the Chromatin immunoprecipitation assays. The bulk of the DNA fragments ranged between 200 and 500 base pairs.
(DOCX)

S2 Fig Immuno-blot of purified DAM proteins. Immuno-blot of purified DAM proteins showing coomassie-stained gel (left) containing GST-tagged and GST-column purified proteins expressed in *E*. *coli*. Gel was blotted and hybridized with 100 μg of anti-DAM1 diluted into 10 ml of TBST. The DAM1 antibody specifically reacted with both GST-tagged DAM1 (the 30kD band) and DAM2 (the 50 kD band) which also contains the target amino acid sequence. Note that that co-purified proteins that eluted with the GST-tagged DAM proteins did not hybridize to the DAM1 antibody, thus indicating some level of specificity. The nature of the antigenic 30 kD band that eluted with the GST-tagged DAM2 protein is unknown, but is likely to be a breakdown product of the DAM2 protein. The level of specificity of the antibody in vivo under non-denaturing conditions is unclear, but given the fact that likely DNA targets of DAM1 can be specifically precipitated with this antibody, it seems likely that it is reactive in vivo.
(DOCX)

S3 Fig FT2 promoter sequence and likely transcription factor binding sites. Above shows *FT2* promoter region and the putative conserved transcription factor binding sites are marked. The pull down DNA fragments mainly enriched in P1, P2 and P5 amplification regions. P1 and P5 amplicons are noted in bold regions, and the P2 region is underlined. Colored boxes indicate possible transcription factor binding sites.
(DOCX)
