# Supplementary material for: Coordinated Expression of FLOWERING LOCUS T and DORMANCY ASSOCIATED MADS-BOX-Like Genes in Leafy Spurge
Source: PLoS One. 2015 May 11;10(5):e0126030. doi: 10.1371/journal.pone.0126030 (PMC4427404; doi:10.1371/journal.pone.0126030)
Supplement: S1 Table — Primers used for qRT-PCR to determine gene expression and chromatin immunoprecipitation result analysis. (DOCX) [file pone.0126030.s001.docx]

| Supplemental Table 1 Primers used for qRT-PCR to determine gene expression and chromatin immunoprecipitation result analysis | | |  |  |
| --- | --- | --- | --- | --- |
| Primer name | Nucleotide sequence (5'-3') | Experimental use | | Size |
| DAM1-F | GAAGACGTGCAAGCATGC | DAM1 specific gene expression | | 186 |
| DAM1-R | CAATTGTCAACTATTTATTGGATGG |  | |  |
| DAM2-F | TGACTCGGGTGATCGAAAG | DAM2 specific gene expression | | 313 |
| DAM2-R | AGTCGCTCGTTCTCTTCC |  | |  |
| FT2-F | GGTATGAGAGTCCAAGACCAGCG | FT2 specific gene expression | | 303 |
| FT2-R | ATTTTCATAGATAATATGTATTACTA |  | |  |
| FT4-F | GGTATGAGAGTCCAAGACCAGCG | FT4 specific gene expression | | 337 |
| FT4-R | CATATATGTAAACAAAAACTTGCAAGGC |  | |  |
| P1-F | AGTAATGTTAATCAATTGGTTTCT | FT2 promoter fragment amplification | | 379 |
| P1-R | CATGTTAAGAGCTAATAGACACCT |  | |  |
| P2-F | ACATTTTTCAAAGGTGTCTATTA | FT2 promoter fragment amplification | | 300 |
| P2-R | GTCCGCCACTGATGGTGAAT |  | |  |
| P3-F | AGGAGGTCAGTCGGATTTGTAGAC | FT2 promoter fragment amplification | | 256 |
| P3-R | GCTCCCAATCTGTTATTCCTTTTT |  | |  |
| P4-F | AAAATAGCTCGTAAGGACTGAAGA | FT2 promoter fragment amplification | | 386 |
| P4-R | ACTCCCCTTAAAGTTTTGTTGTTA |  | |  |
| P5-F | AAAAATGAGTAAGGCTCCGTTCTT | FT2 promoter fragment amplification | | 286 |
| P5-R | TATGCCATTTTTCTTCGAGATAGG |  | |  |
| SAND-F | AGGGCAACACAGACTACAAAATG | Internal control for gene expression | | 298 |
| SAND-R | ATAAAGTTCGAAATCTGGGGTGAC |  | |  |
