# Supplementary material for: Coordinated Expression of FLOWERING LOCUS T and DORMANCY ASSOCIATED MADS-BOX-Like Genes in Leafy Spurge
Source: PLoS One. 2015 May 11;10(5):e0126030. doi: 10.1371/journal.pone.0126030 (PMC4427404; doi:10.1371/journal.pone.0126030)
Supplement: S2 Table — List of transcripts assembled from three different expression analyses of leafy spurge including two containing dormant and growing bud samples that were identified as having sequence similarity (E values < 10E-5) with DAM1 in a BlastX search of the transcriptome database. Highlighted contigs are from genes that have good levels of expression (> 100 transcripts per million) in ecodormancy. (DOCX) [file pone.0126030.s002.docx]

Supplemental Table 2: List of transcripts assembled from three different expression analyses of leafy spurge including two containing dormant and growing bud samples that were identified as having sequence similarity (E values < 10E-5) with DAM1 in a BlastX search of the transcriptome database. Highlighted contigs are from genes that have good levels of expression (> 100 transcripts per million) in ecodormancy.

| Transcript | Arabidopsis homologue | | Target sequence | Target sequence homology |
| --- | --- | --- | --- | --- |
| DAM1 | AT4G24540 | AGL24 | RRGLFKKAHELS | RRGLFKKAHELS |
| comp137427_c0_seq79 | AT4G24540 | AGL24 | RRGIFKKAHELS | RRG+FKKAHELS |
| comp102355_c0_seq13 | AT4G24540 | AGL24 | RRGIFKKAHELS | RRG+FKKAHELS |
| comp111608_c0_seq1 | AT2G22540 | SVP | RRGLFKKAEELA | RRGLFKKA EL+ |
| comp110466_c0_seq1 | AT2G22540 | SVP | RRGLFKKAEELA | RRGLFKKA EL+ |
| comp68910_c0_seq1 | AT3G57390 | AGL18 | RNGLLKKAKELS | R GL KKA ELS |
| comp116318_c0_seq4 | AT2G14210 | AGL44 | RNGLLKKAKELS | R GL KKA ELS |
| comp135076_c1_seq1 | AT2G14210 | AGL44 | RSGLLKKAKELS | R GL KKA ELS |
| comp132989_c0_seq38 | AT4G11860 | Unknown | RNGLLKKAYELS | R GL KKA+ELS |
| comp122790_c2_seq3 | AT4G11880 | AGL14 | RNGLLKKAYELS | R GL KKA+ELS |
| comp100007_c0_seq1 | AT5G15800 | SEPALLATA1 | RNGLLKKAYELS | R GL KKA+ELS |
