# Supplementary material for: Coordinated Expression of FLOWERING LOCUS T and DORMANCY ASSOCIATED MADS-BOX-Like Genes in Leafy Spurge
Source: PLoS One. 2015 May 11;10(5):e0126030. doi: 10.1371/journal.pone.0126030 (PMC4427404; doi:10.1371/journal.pone.0126030)
Supplement: S1 Fig — Ethidium stained agarose gel showing the size range of the sonicated and precipitated DNA used for the Chromatin immunoprecipitation assays. The bulk of the DNA fragments ranged between 200 and 500 base pairs. (DOCX) [file pone.0126030.s003.docx]

Supplemental Figure
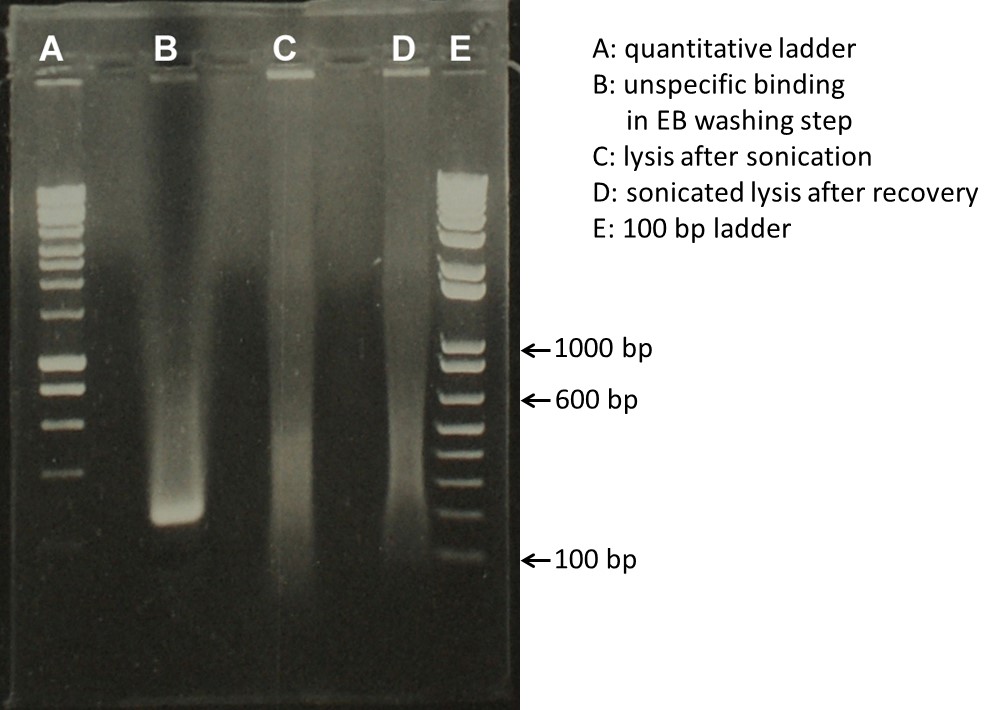
1: Ethidium stained agarose gel showing the size range of the sonicated and precipitated DNA used for the Chromatin immunoprecipitation assays. The bulk of the DNA fragments ranged between 200 and 500 base pairs.
