# Supplementary material for: Coordinated Expression of FLOWERING LOCUS T and DORMANCY ASSOCIATED MADS-BOX-Like Genes in Leafy Spurge
Source: PLoS One. 2015 May 11;10(5):e0126030. doi: 10.1371/journal.pone.0126030 (PMC4427404; doi:10.1371/journal.pone.0126030)
Supplement: S2 Fig — Immuno-blot of purified DAM proteins showing coomassie-stained gel (left) containing GST-tagged and GST-column purified proteins expressed in E. coli. Gel was blotted and hybridized with 100 μg of anti-DAM1 diluted into 10 ml of TBST. The DAM1 antibody specifically reacted with both GST-tagged DAM1 (the 30kD band) and DAM2 (the 50 kD band) which also contains the target amino acid sequence. Note that that co-purified proteins that eluted with the GST-tagged DAM proteins did not hybridize to the DAM1 antibody, thus indicating some level of specificity. The nature of the antigenic 30 kD band that eluted with the GST-tagged DAM2 protein is unknown, but is likely to be a breakdown product of the DAM2 protein. The level of specificity of the antibody in vivo under non-denaturing conditions is unclear, but given the fact that likely DNA targets of DAM1 can be specifically precipitated with this antibody, it seems likely that it is reactive in vivo. (DOCX) [file pone.0126030.s004.docx]

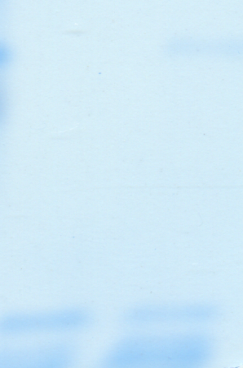

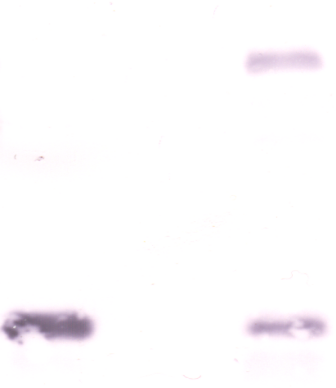

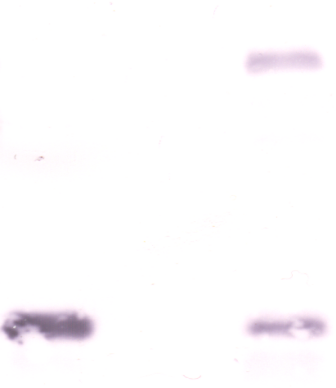


GST-DAM1

GST-DAM1

GST-DAM2

GST-DAM2

kD

50

30

SDS Gel

Immuno-blot

Supplemental Figure 2: Immuno-blot of purified DAM proteins showing coomassie-stained gel (left) containing GST-tagged and GST-column purified proteins expressed in E. coli. Gel was blotted and hybridized with 100 µg of anti-DAM1 diluted into 10 ml of TBST. The DAM1 antibody specifically reacted with both GST-tagged DAM1 (the 30kD band) and DAM2 (the 50 kD band) which also contains the target amino acid sequence. Note that that co-purified proteins that eluted with the GST-tagged DAM proteins did not hybridize to the DAM1 antibody, thus indicating some level of specificity. The nature of the antigenic 30 kD band that eluted with the GST-tagged DAM2 protein is unknown, but is likely to be a breakdown product of the DAM2 protein. The level of specificity of the antibody in vivo under non-denaturing conditions is unclear, but given the fact that likely DNA targets of DAM1 can be specifically precipitated with this antibody, it seems likely that it is reactive in vivo.
