# Supplementary material for: Coordinated Expression of FLOWERING LOCUS T and DORMANCY ASSOCIATED MADS-BOX-Like Genes in Leafy Spurge
Source: PLoS One. 2015 May 11;10(5):e0126030. doi: 10.1371/journal.pone.0126030 (PMC4427404; doi:10.1371/journal.pone.0126030)
Supplement: S3 Fig — Above shows FT2 promoter region and the putative conserved transcription factor binding sites are marked. The pull down DNA fragments mainly enriched in P1, P2 and P5 amplification regions. P1 and P5 amplicons are noted in bold regions, and the P2 region is underlined. Colored boxes indicate possible transcription factor binding sites. (DOCX) [file pone.0126030.s005.docx]

atattcatcaaaattaaaaaatatctttagtatcatgcatgccatgcatagagagcaaatgcctgaatatatagatcacta**agtaatgttaatcaattggtttctaaatttaggaatatcttgtttgctcactgaattgtcaaaaaattaattcaactaaatttttatactaatagttaaagttcaagaacactttttactgttctcacatgaaaatttcatgatgggttttaagtgtgtataacacatgactcatcccatcatcaccatgtattcaaattcaatcagtagatgcagttgatcatattctaattctggttgtttggtctggaatgttttaatactatgttaaaaaaaacaattaaactaaaagtttaattggttaattataaaagttcaagaaaataacttttattatattcttga**catttttcaaaggtgtctattagctcttaacatgcatatagtgattattaaccttttaacttatttaaaatgacatgtattcaattttttatgatcctattggattaatcatgtcacttaataatatcactaatttattgcattgaagtggctaatgaaacactctataaatttaggaggtcagtcggatttgtagaccaaattcaagaatatcgattataacataaatgttcttttgcatatgtatcacaggtccatccaaccatgagtttttttaattcaccatcagtggcggacccaggatttgagttttgggggggctaattgtagccttttatttcggattttttttatccttgtatgtaaaaatatttaaaagtaaactatataaaattttaattgtttcaaaaaggaataacagattgggagctaaattcttaattactgatataattagaataagaaattggaagagtcaatttttaaaatagctcgtaaggactgaagaattatttttttcagtttcttgtataaggaaaaaaatgaggccataatatttttgaaaaatagatttgaaataataattatagttattttcatttcaaataggggagtaataaaattgaagaattaaaaaagttggaagagcaaatatatagtagcttaggttttagaaaaaaattggagaagtaaatagatagtggtatgggttttagaaaaaaaagagtaagaaaaatgcaaaaaattcaaaggtaaggatagaacctccaaccacttgcataaaactccactacttaaccattcatgctcaacatcatttgtgtttagtatttgcatttggatgtataacaacaaaactttaaggggagtcacccaactacactatagtaaatttttttttatcgaagcggacccaggctggagcccaccccaacccatacttgggtccacccctgttcaccatacatgaggttgtcaagacttgaaaatatgtgtaatatactttatattaatctctgaaaatatgtgtaatatactttattataaaattgttaattaatttaatgaaactaaa**aaaatgagtaaggctccgttcttttttactgaattgaactgaactgaattattaactgaatgaatgtaaattttattgaatttgttgaattgaactgaattgaactgaactgaattgaattgaattgaactgaactgaattgaactgaattgaactgaaatgtgttgaatataagttaaaaagaatagagcctaaaaagaaaagagaatgagactagcgcattatcttaaattggttgtcaatttgtagattcagaacctcc**ctatctcgaagaaaaatggcatacaaacatatataaaatctctaagaaagccagattctcttatatattttgtttttatttttatttatttttattttttaaaagtaggacagtgtaccacaaaatggtataaatataaacaccaagtgaagtgatgatacatagtgaaaataagagagagagaa

ARR binding site Unknown but conserved

ABA responsive CO binding sites?

Myb binding site CaRG box

Supplemental Figure 2:

Above shows *FT2* promoter region and the putative conserved transcription factor binding sites are marked. The pull down DNA fragments mainly enriched in P1, P2 and P5 amplification regions. P1 and P5 amplicons are noted in bold regions, and the P2 region is underlined. Colored boxes indicate possible transcription factor binding sites.
